# Immunophenotyping of Monocyte Migration Markers and Therapeutic Effects of Selenium on IL-6 and IL-1β Cytokine Axes of Blood Mononuclear Cells in Preoperative and Postoperative Coronary Artery Disease Patients

**DOI:** 10.3390/ijms24087198

**Published:** 2023-04-13

**Authors:** Max Wacker, Anna Ball, Hans-Dietmar Beer, Ingo Schmitz, Katrin Borucki, Faranak Azizzadeh, Maximilian Scherner, George Awad, Jens Wippermann, Priya Veluswamy

**Affiliations:** 1 Heart Surgery Research, Department of Cardiothoracic Surgery, Otto-von-Guericke University Hospital, Leipziger Straße 44, 39120 Magdeburg, Germany; max.wacker@med.ovgu.de (M.W.); med.anna.ball@gmail.com (A.B.); faranak.azzizadeh@med.ovgu.de (F.A.); maximilian.scherner@med.ovgu.de (M.S.); george.awad@med.ovgu.de (G.A.); jens.wippermann@med.ovgu.de (J.W.); 2 Department of Dermatology, University Hospital Zurich, CH-8952 Schlieren, Switzerland; hans-dietmar.beer@usz.ch; 3 Department of Molecular Immunology, Medical Faculty of Ruhr-University Bochum, 44801 Bochum, Germany; ingo.schmitz@ruhr-uni-bochum.de; 4 Institute of Clinical Chemistry and Pathobiochemistry, Otto-von-Guericke University, 39120 Magdeburg, Germany; katrin.borucki@med.ovgu.de

**Keywords:** selenium, post-cardiotomy syndrome, coronary artery disease, pro-inflammatory cytokines and monocytes

## Abstract

Multivessel coronary artery disease (CAD) is characterized by underlying chronic vascular inflammation and occlusion in the coronary arteries, where these patients undergo coronary artery bypass grafting (CABG). Since post-cardiotomy inflammation is a well known phenomenon after CABG, attenuation of this inflammation is required to reduce perioperative morbidity and mortality. In this study, we aimed to phenotype circulating frequencies and intensities of monocyte subsets and monocyte migration markers, respectively, and to investigate the plasma level of inflammatory cytokines and chemokines between preoperative and postoperative CAD patients and later, to intervene the inflammation with sodium selenite. We found a higher amplitude of inflammation, postoperatively, in terms of CCR1^high^ monocytes and significantly increased pro-inflammatory cytokines, IL-6, IL-8, and IL-1RA. Further, in vitro intervention with selenium displayed mitigating effects on the IL-6/STAT-3 axis of mononuclear cells derived from postoperative CAD patients. In addition, in vitro selenium intervention significantly reduced IL-1β production as well as decreased cleaved caspase-1 (p20) activity by preoperative (when stimulated) as well as postoperative CAD mononuclear cells. Though TNF-α exhibited a positive correlation with blood troponin levels in postoperative CAD patients, there was no obvious effect of selenium on the TNF-α/NF-κB axis. In conclusion, anti-inflammatory selenium might be utilized to impede systemic inflammatory cytokine axes to circumvent aggravating atherosclerosis and further damage to the autologous bypass grafts during the post-surgical period.

## 1. Introduction

Multivessel coronary artery disease (CAD) accounts for 17.8 million annual deaths and is the third leading cause of mortality, globally [1]. CAD is mainly characterized by an underlying chronic inflammation due to the presence of densely accumulated atherosclerotic plaques causing occlusion in the lumen of two or more coronary arteries [2]. These plaques are largely composed of fatty streaks with lipid-laden macrophages, which are infiltrated T lymphocytes that are further responsible for a dysfunctional coronary artery endothelium [3] and sustainment of progressive inflammation [2,4]. Here, the localized inflammation associated with atherosclerotic plaques produces a plethora of inflammatory cytokines, including Type 1 [5], Type 2 (especially IL-4) [6], and Type 3 immunity associated cytokines [7], spilling into the circulation [8,9]. Compelling evidence has shown the association of TNFα, IL-1β, MCP-1, MIP-1a, and eotaxin with CAD pathogenesis [10,11]. Of note, serum IL-6 has been regarded as a highly predictive marker for CAD, when screened among an intermediate cardiovascular risk population [12]. In addition to IL-6, an elevated serum level of the IL-1 receptor antagonist (IL-1RA) is also considered to be a strong independent predictor for CAD mortality [13,14]. These inflammatory cytokines further activate monocytes [15] to perpetuate systemic inflammation [16]. As subgrouping of blood circulating monocytes has been widely acknowledged since 2010, based on the cell surface expression of CD14 and CD16, dictating into (i) classical monocytes (CD14^high^CD16^dim^), (ii) intermediate monocytes (CD14^Int^CD16^Int^), and (iii) non-classical monocytes (CD14^dim^CD16^high^) [17,18], there were several reports demonstrating increased numbers of circulating inflammatory monocytes in association with plaque progression [19], sustaining coronary atherosclerosis [20,21] and CAD prevalence [22]. Further, these inflammation-seeking classical monocytes traffic towards atherosclerotic lesions, which is mainly dependent on the CCR2 migration marker [23]. Furthermore, the experimental model has supported the notion of monocyte influx through increased expressions of migration markers, CCR1 and CCR5, into the atherosclerotic arteries [24].

Though optimal medical therapy exists to curb progressive atherosclerosis in CAD [25,26], a majority of these multivessel CAD patients undergo surgical intervention, popularly known as “coronary artery bypass grafting (CABG).” CABG is a gold standard method, where utilization of a heart lung machine exemplifies a standard procedure in traditional cardiac surgery [27,28]. During surgery, the contact of circulating ex vivo blood with non-physiological surfaces additionally exacerbates the degree of inflammation, which might be highly vulnerable for high-risk group comorbid CAD patients with renal failure and diabetes [29,30]. CAD patients who underwent on-pump bypass surgery exhibited increased systemic levels of (i) circulating neutrophils, (ii) neutrophil elastase [31], (iii) complement components (C3a and C5a) [32], (iv) platelet activating factors [33], (v) C-reactive protein (CRP) (an acute phase reactant), (vi) pro-inflammatory cytokines, TNF-α, IL-6, IL-8 [34], (vii) adhesion molecules (E-selectin, P-selectin, ICAM-1) [35], and cardiac troponin I [36,37,38]. These findings demand a better understanding of complex CAD pathogenesis for further interventional aspects during perioperative and/or postoperative cardiac bypass surgery. 

Selenium is an essential trace mineral that is crucial for cellular protection against free radicals (oxidative stress elements) and inflammation [39]. Several studies have noted decreased serum concentrations of selenium in cardiovascular diseases, including in CAD patients [40,41]. Concomitantly, selenium deficiency was shown to be associated with an increased risk for cardiovascular diseases, where a 50% increase in selenium levels was associated with 24% reduced risk for CAD [41]. Upon increased selenium supplementation, meta-analysis data have demonstrated decreased serum levels of CRP and triglycerides, which are known risk factors for CAD [42]. In fact, selenium deficit has also reduced the coronary flow reserve which is crucial for the development of atherosclerosis [43]. Clinical trials have been conducted to restore selenium levels through administration of high-dose selenium, where selenium exhibited reduced mortality under the state of systemic inflammatory syndrome [44,45]. However, none of the studies demonstrate the effect of selenium on pro-inflammatory cytokine axes at its molecular level among preoperative and postoperative CAD patients. We therefore aimed to assess the level of chronic inflammation, particularly with mononuclear cells and cytokine axes, in both preoperative and postoperative CAD patients and further, to intervene (in vitro) with selenium (as sodium selenite) to observe its beneficial effects between preoperative and postoperative CAD cohorts.

## 2. Materials and Methods

### 2.1. Patients and Controls

A total of 76 CAD patients (preoperative and postoperative) were recruited at the Department of Cardiothoracic Surgery at Otto-von-Guericke University Hospital Magdeburg, under the approval of the Institutional Ethics Committee of the medical faculty of Otto-von-Guericke University in Magdeburg (study approval number: 18/19). All patients were diagnosed with coronary artery disease using coronary catheterization (angiogram) and were directed to CABG surgery by the decision of the local heart team. CAD disease stages and Euroscore II (%) have been summarized for all CAD patients (Table 1). The exclusion criteria for CAD patients were known infections, such as hepatitis and HIV, cancers, hemodynamic instability, and anemia. All recruited CAD patients were taking medications and were comorbid with several underlying diseases, such as type 2 diabetes, arterial hypertension, and chronic kidney disease. Furthermore, 10 younger (≤40 years) and 10 older (≥50 years) normal controls were recruited in our study. Saphenous veins during CABG surgery were collected from four CAD patients (Table 1 and Table 2). All subjects gave written informed consent. The basic and clinical characteristics of the CAD patients as well as the basic characteristics of normal controls are outlined in Table 1 and Table 2, respectively. The treatments given before and after cardiac surgery in the preoperative as well as postoperative CAD cohorts are summarized in Table 3.

Anesthetic induction was performed with propofol, sufentanyl, and rocuronium. Maintenance of anesthesia was performed with inhaled anesthetics and sufentanyl. All patients received perioperative antibiotic therapy with cefuroxime. Perioperative catecholamine therapy was routinely performed with dobutamine and norepinephrine, and Buckberg cardioplegia was used as the cardioplegic solution in all cases. To achieve the necessary blood anticoagulation under cardiopulmonary bypass, unfractionated heparin was given intravenously, which was antagonized by protamine at the end of surgery.

Postoperatively, all patients were rapidly extubated and routinely received potassium and magnesium infusions. Postoperative pain management was performed with oxycodone and metamizole. Furthermore, the existing preoperative regular medications (listed in Table 3) were routinely re-introduced within three postoperative days. Thromboprophylaxis was subcutaneously performed with unfractionated heparin. 

### 2.2. Blood Collection, Plasma Isolation, and PBMC Culture

20 mL of peripheral blood was collected into heparinized tubes from CAD patients, both the younger and older normal controls. Cellular fractions, such as peripheral blood mononuclear cells (PBMCs), were isolated using the ficoll-hypaque gradient technique. These PBMCs were either used for flow cytometric analysis and/or cultured for all functional assays to evaluate the therapeutic effects of selenium. In addition, plasma was collected by centrifuging 10 mL of blood that was collected into heparinized tubes from all subjects. Cell culture supernatants were also collected for functional assays. Both plasma and cell culture supernatant were aliquoted and stored at −80 °C and were thereby further used for Luminex.

### 2.3. Selenium Concentration and Treatment

Selenium (as sodium selenite, Sigma Aldrich, St. Louis, MO, USA) was used to intervene in the inflammatory analytes. An initial pharmacokinetic study with 8 different selenium concentrations (50 nM, 100 nM, 500 nM, 1 µM, 5 µM, 10 µM, 20 µM, and 50 µM), was performed to select the interventional concentrations (100 nM and 5 µM) of selenium that were used throughout the study, which represents rather physiologically therapeutic doses. Here, human leukemia monocytic cell lines (THP-1) (ACC No.16) (German Collection of Microorganisms and Cell Cultures (DSMZ), Braunschweig, Germany), were treated with 8 different concentrations of selenium in the presence of lipopolysaccharides (LPS) (*E. coli* O26:B6) (L2654, Sigma). The cell pellets were stored in RLT lysis buffer with 1% β-mercaptoethanol (M6250, Sigma Aldrich) for further RNA (*IL1B* gene expression) studies (Figure S1). About 1–3 × 10^6^ PBMCs were treated with two different concentrations (100 nM and 5 µM) of selenium for 24 h and were subsequently treated with 100 ng of rIL-6 (Peprotech) for the last 30 min. The cells were trypsinized and stored for the detection of phospho-STAT-3 activity by Western blotting. Furthermore, 1–3 × 10^6^ PBMCs were treated with two different concentrations (100 nM and 5 µM) of selenium for 24 h and were subsequently treated with LPS (100ng/mL) for 16 h and further treated with Nigericin (5 µM) for 2 and 6 h. The cell supernatant was stored for IL-1β by Luminex and a subsequent cleaved caspase-1 (p20) by Western blotting. Likewise, 1–3 × 10^6^ PBMCs were treated with two different concentrations (100 nM and 5 µM) of selenium for 24 h and were subsequently treated with LPS (100 ng/mL or 1 µg/mL) for 1 to 3 h. The cell pellets were trypsinized and stored for the detection of p-P65 activity. Similar treatment conditions were adapted for treating 1–2 × 10^6^ human vascular endothelial cells to detect phospho-STAT-3 or phospho-P65 activities.

### 2.4. Quantification of IL1B Gene Expression by Real-Time PCR

The concentration and purity of the eluted RNA, obtained from pharmacokinetic study for selecting the selenium concentration, were analyzed using a Nanophotometer (Implen). An equal concentration of the RNA template (1 µg) was reverse-transcribed with random primers using the Maxima first strand cDNA synthesis kit with dsDNase (catalog # K1671, ThermoFischer Scientific, Waltham, MA, USA),) in order to achieve the initial adjustment of sample material, leading to comparable cDNA concentrations in each reverse transcription reaction; the cDNA was stored in −80 °C until usage. Quantitative (q) PCR was performed using the components: (i) cDNA template, (ii) primers (forward and reverse), and (iii) PowerUp SYBR Green Master mix (Applied Biosystems, Waltham, MA, USA, catalog # A25741) with qPCR grade nuclease-free water in the volume of 25 µL. The instrument used for qPCR was the Applied Biosystems StepOnePlus real-time PCR system, which was installed with StepOne Software (Version 2.1). Samples were run for 40 cycles (95 °C for 15 s, 60 °C for 30 s, and 72 °C for 30 s). All samples were run in duplicates. No template control (NTC) served as negative controls. Here, β2-microglobulin *(B2M)* was used as an internal housekeeping gene (reference gene). Calculation was performed using Ct values, averaged from duplicate measurements. The relative expression of a target transcript within a given sample was calculated using the delta (Δ) Ct method as previously described [46]. The sequences of the primers used in this study are (i) *IL1B* (forward: TACCTGTCCTGCGTGTTGAA and reverse: TCTTTGGGTAATTTTTGGGATCT) and (ii) *B2M* (forward: TTCTGGCCTGGAGGCTATC and reverse: TCAGGAAATTTGACTTTCCATTC).

### 2.5. Saphenous Vein Collection and Primary Vascular Endothelial Cell (pVECs) Isolation

A leftover piece of saphenous vein was collected from CAD patients during coronary artery bypass graft surgery (CABG). For pVECs isolation, the veins were flushed with PBS to remove blood debris and were consequently filled with 0.4% Collagenase A solution (Sigma-Aldrich, St. Louis, MI, USA) and incubated at 37 °C for 30 min. By rinsing with PBS, the cell suspension was collected and plated on 0.1% gelatin-coated cell culture flasks using a cell-specific growth medium (Endothelial Cell Growth Medium C22110, Promocell, Heidelberg, Germany) containing 1% penicillin-streptomycin solution and 10% fetal calf serum (FCS) with a density of 0.6–1.0 × 10^5^ cells/cm^2^ (as passage 0), as previously described [47]. We cultured these primary endothelial cells up to the 10th passage and thereafter, the cells were stored at −150 °C (50% FCS, 10% DMSO). These cells were used for all functional assays involving the evaluation of the therapeutic effects of selenium.

### 2.6. Blood Monocytes and pVEC Phenotyping by Flow Cytometry

In order to phenotype monocyte subsets and associated migration markers, freshly obtained PBMCs from CAD patients and normal controls were stained with fluorochrome coupled antibodies against the cell surface markers, CD14-FITC (clone HCD14, BioLegend, San Diego, CA, USA), CD16-APC (clone 3G8, BioLegend), CCR2-PE (clone K036C2, BioLegend), CX_3_CR1-BV421 (clone 2A9-1, BioLegend), CCR5-BV711 (clone J418F1, BioLegend), and CCR1-PeCy7 (5F10B29, BioLegend). We used respective isotype controls, such as APC-mouse IgG1, (clone MOPC-21, BioLegend), PE-Mouse IgG2b (clone MOPC-173, BioLegend), BV421-Rat IgG2b (clone RTK 4530, BioLegend), BV711-Mouse IgG2b (clone RTK 4530BioLegend), and PECy7-Mouse IgG1 (clone MOPC-21, BioLegend). Prior to the staining procedure, the cells were washed and stained for live/dead dye using a Zombie Aqua fixable viability kit (BioLegend) and were subsequently blocked for the Fc receptor using human Trustain FcX (BioLegend). Single stain controls were employed using mouse and rat compensation beads (BioLegend). As a post-staining procedure, the cells were washed and finally fixed with 1% paraformaldehyde. Likewise, the pVECs were stained with endothelial cell related markers, CD31-BV421 (WM59, BioLegend), Von Willebrand Factor (vWF)—Alexa Fluor 488 (EPSISR15, Abcam, Cambridge, UK), ICOS-L-PE (2D3, BioLegend), and PD-L1-APC (29E.2A3). All sample acquisitions were performed using flow cytometry (LSR II Fortessa, BD Biosciences), installed with FACS Diva software (version 6.1.3) (BD Biosciences, Franklin Lakes, New Jersey, USA). Further, analysis was performed using FlowJo version 9.9.6 (Tree star, Inc. Ashland, OR, USA).

### 2.7. Array of Cytokines, Chemokine and Growth Factor Measurement by Luminex

We used Luminex xMAP technology for multiplexed quantification of 15 human cytokines, chemokines, and growth factors. The multiplexing analysis was performed using the Luminex™ 200 system (Luminex, Austin, TX, USA) by Eve Technologies Corp. (Calgary, AB, Canada). Fifteen markers were simultaneously measured in the samples using Eve Technologies’ Human Cytokine Pro-inflammatory Focused 15-Plex Discovery Assay^®^ (Millipore Sigma, Burlington, MA, USA) according to the manufacturer’s protocol. The 15-plex consisted of GM-CSF, IFN-γ, IL-1β, IL-1RA, IL-2, IL-4, IL-5, IL-6, IL-8, IL-10, IL-12(p40), IL-12(p70), IL-13, MCP-1, and TNF-α. Assay sensitivities of these markers range from 0.1–9.5 pg/mL for the 15-plex. The assay was performed by Eve Technologies, Canada.

### 2.8. Detection of Phosphorylation of STAT-3 and P65 and Cleaved Caspase-1 (p20 Subunit) by Western Blot

Isolated 1–3 × 10^6^ PBMCs and 0.5–1 × 10^6^ human vascular endothelial cells were pelleted and treated with homemade lysis buffer [48]. This is succeeded by measuring the protein concentration of the samples at 562 nm with a spectrometer (Tecan, Megelan, using Pierce^TM^ BCA Protein Assay Kit (Cat#23225, Thermo Fischer Scientific), where 20 µg of sample protein was subjected to SDS page electrophoresis and transferred to the PVDF membrane according to the standard laboratory procedure. We employed human primary antibodies for phospho-STAT-3 (Tyr 705) (D3A7), STAT-3 (D3Z2G), phospho-P65 (93H1), P65 (D14E12) (1:1000 or 1:2000), and cleaved caspase-1 (p-20) (Bally-1, Adipogen Life Sciences, San Diego, CA, USA) to detect the respective proteins. We used α-Tubulin (11H10) (Cell Signaling, Danvers, MA, USA) and/or β-actin (2D4H5) (Proteintech, Rosemont, IL, USA) as the loading protein and anti-rabbit IgG, HRP-linked antibody (Cat# 7074, Cell Signaling), and AP-conjugated anti-mouse IgG (S3721, Promega, Madison, WI, USA,) were used as secondary antibodies. As substrate, we used Pierce^TM^ ECL Western, Super Signal^TM^ West Femto, or BCIP/NBT (Promega). The subsequent development of bands was detected using an INTAS ECL ChemoStar Imager (INTAS science Imaging) and further densitometries were quantified using LabImage ID software.

### 2.9. In Vitro Dynamic Analysis Using the Chandler Loop System

To simulate extracorporeal circulation, a hemodynamic assembly was used to study the effect of selenium on CAD mononuclear cells and cytokine levels. For this purpose, we used a modified Chandler loop model as described by Fink et al. [49]. Briefly, polyvinyl chloride (PVC) tubes with an inner diameter of 5.0 mm and a wall thickness of 1.0 mm (VWR #228–1752) were fixed with external connectors to form loops, with a total length of 500 mm (50 cm). The loops were then secured on a customized rack with a motor unit and rotation speed control (used and rotated in a water bath as shown in our previous study [50]). The Chandler loop system was obtained directly from the product developer (Ebo Kunze, Industriedesign, Neuffen, Germany). In this study, 5 mL of peripheral blood from pre-operative CAD patients was collected and loaded into PVC tubes, with/without the addition of a therapeutic concentration of selenium (1 µg for 5 mL blood) [45] and was incubated for 6, 12, and 24 h. Upon incubation, the PVC tubes were cut into pieces and were trypsinized to retrieve bound monocytes and were then subjected to the ficoll-hypaque method to isolate the cellular fractions (PBMCs) as well as plasma for monocyte marker and cytokine analysis.

### 2.10. Statistical Analysis

The differences between the monocyte frequencies and intensities of monocyte migration markers and soluble analytes in CAD patients and controls were performed using the nonparametric Kruskal–Wallis Test with Dunn’s post hoc tests. During comparison between two variables, a nonparametric Mann–Whitney *U*-test or unpaired student *t*-test was used. The correlation between two different variables, either between clinical parameters and cytokines or a CCR1 monocyte migration marker, was analyzed using nonparametric Spearman’s correlation coefficient tests. The *p* value (*p* < 0.05) was considered significant in the study. The software used for graphical representations was GraphPad Prism version 8.0.1.

## 3. Results

### 3.1. Differential Expression of Monocyte Migration Markers between Preoperative and Postoperative CAD Patients

**Figure 1:**
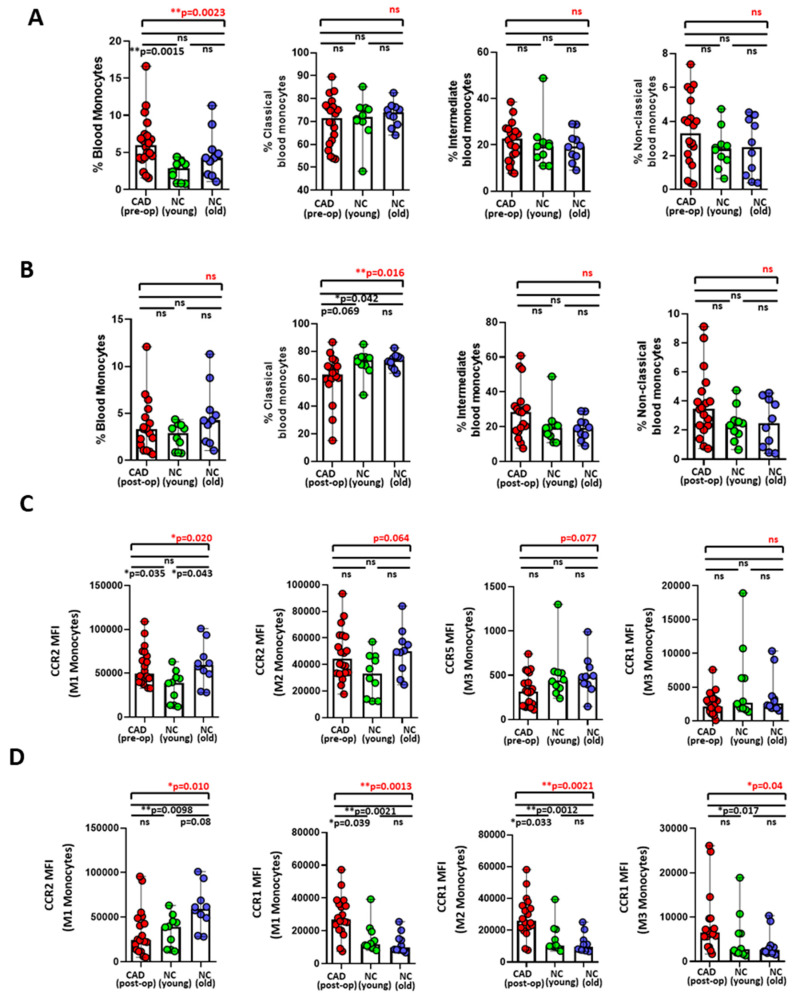
Frequencies of blood total monocytes and subsets and intensities of monocyte migration markers among preoperative and postoperative CAD patients and controls. Comparison of the frequencies of blood monocytes and frequencies of monocyte subsets (classical, intermediate, and non-classical) in PBMCs of (**A**) pre-operative CAD patients and younger and older controls (upper panel), (**B**) post-operative CAD patients and younger and older controls (lower panel). (**C**) Comparison of median fluorescent intensities (MFI) of expressed monocyte migrations markers (CCR2, CCR5, and CCR1) in the classical (M1), intermediate (M2), and non-classical (M3) monocyte subsets of preoperative CAD patients and younger and older controls (upper panel). (**D**) Comparison of the MFI of expressed monocyte migrations markers (CCR2 and CCR1) in M1, M2, and M3 monocyte subsets of postoperative CAD patients and younger and older controls (lower panel). Statistical analyses were performed by a non-parametric Kruskal–Wallis test (overall differences between analyzed groups in red) with Dunn’s multiple comparison test or a Mann–Whitney test (differences between each group in black). The colored dot plots represent individual data values. The vertical lines in the scatter dot plot with bar represents the median with range. (* *p* ≤ 0.05; ** *p* ≤ 0.01; ns: non-significant).

PBMCs obtained from 20 preoperative and 18 postoperative CAD patients and 10 younger and 10 older normal controls were phenotyped for blood monocyte subsets and the intensities of migration markers by flow cytometry. Here, we observed significantly increased frequencies of blood-circulating monocytes in preoperative CAD patients when compared with the younger normal controls (Figure 1A), but not with the older controls. We did not observe any significant differences in the frequencies of monocytes between the postoperative CAD patients and younger or older controls (Figure 1B). Further, upon classifying these monocytes into classical (M1), intermediate (M2), and non-classical subsets (M3), based on the intensities of cell surface markers CD14 and CD16 (Figure S1B), we observed a significant decrease in the frequencies of classical monocytes among postoperative CAD patients when compared with older controls (Figure 1B), whereas the differences in monocyte subsets were not evident between the preoperative CAD patients and control groups (Figure 1A).

Among the four investigated monocyte migration markers, CCR2, CX_3_CR1, CCR5, and CCR1, we strikingly found that the intensities of CCR1 were significantly increased in the M1, M2, and M3 subsets of monocytes in postoperative CAD patients when compared with older controls, as well as with younger controls in terms of M1 and M2 (Figure 1D). Such differences with CCR1 intensities were not evident between preoperative CAD patients and normal controls (Figure 1C). Of note, CCR1 intensities were significantly increased among postoperative CAD patients in comparison to preoperative CAD patients (Figure S1D). Further, CCR2 intensities were significantly decreased in M1 monocytes among postoperative CAD patients when compared with older normal controls (Figure 1D), as well as with preoperative CAD patients (Figure S1D). Furthermore, CCR2 intensities on the M1 and M2 monocytes exhibited increased tendencies when compared with young controls (Figure 1C). Though not reaching the level of statistical significance, CCR5 intensities in M3 monocytes were markedly reduced among preoperative CAD patients when compared with the controls (Figure 1C). Likewise, we observed a significant reduction in the CX_3_CR1 intensities in M1 monocytes among postoperative patients when compared with the younger controls (Figure S1C).

### 3.2. Increased Plasma Levels of Inflammatory Cytokines among Postoperative CAD Patients

**Figure 2:**
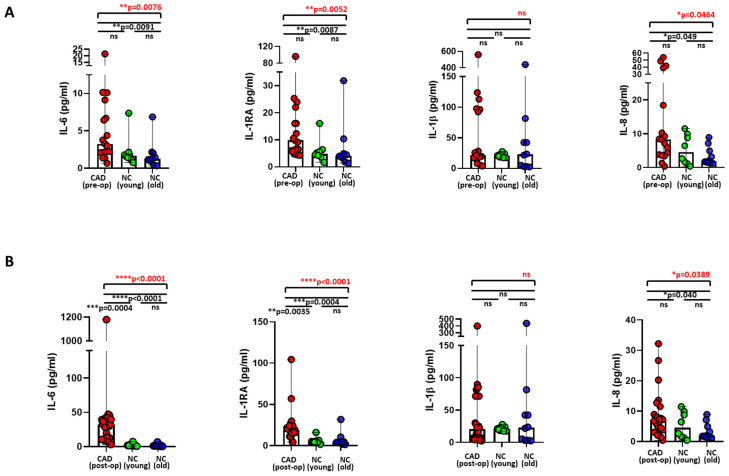
Plasma levels of pro-inflammatory cytokines among preoperative and postoperative CAD patients and controls. **(A**) Comparison of plasma IL-6, IL-1RA, IL-1β, and IL-8 levels between preoperative CAD patients and younger and older controls (upper panel), (**B**) Comparison of plasma IL-6, IL-1RA, IL-1β, and IL-8 levels between postoperative CAD patients and younger and older controls (lower panel). Statistical analyses were performed by a non-parametric Kruskal–Wallis test (overall differences between analyzed groups in red) with Dunn’s multiple comparison test (differences between each group in black). The colored dot plots represent individual data values. The vertical lines in the scatter dot plot with bar represents the median with range. (* *p* ≤ 0.05; ** *p* ≤ 0.01; *** *p* ≤ 0.001; **** *p* ≤ 0.0001; ns: non-significant).

Plasma samples obtained from 17 preoperative and 24 postoperative CAD patients and eight younger and 10 older normal controls were determined for array of cytokines and chemokines. Strikingly, we observed significantly elevated plasma levels of pro-inflammatory cytokines, IL-6 and IL-8, among preoperative CAD patients when compared with the older normal controls (Figure 2A). Of note, the IL-1R antagonist (IL-1Ra) was also significantly increased among preoperative CAD patients when compared with the older normal controls (Figure 2A). Likewise, we observed significantly elevated plasma levels of IL-6 and IL-8 among postoperative CAD patients when compared with both younger and older normal controls, respectively (Figure 2B). Similarly, IL-1RA cytokine was also significantly increased among postoperative CAD patients when compared with both younger and older normal controls (Figure 2B). Of note, the amplitudes of IL-6 and IL-1RA cytokines were significantly elevated among postoperative CAD patients when compared with preoperative CAD patients (Figure S2B). Plasma levels of cytokines, IL-5, IL-4, and IL-10, were also significantly elevated among postoperative CAD patients when compared with the preoperative patient group (Figure S2B). However, interestingly, plasma GM-CSF (granulocyte-macrophage colony stimulating factor) was significantly reduced among postoperative CAD patients when compared with the preoperative CAD patients (Figure S2B). Whereas we observed only elevated plasma TNFα levels among postoperative CAD patients when compared with younger controls, we did not observe differences in the levels of TNFα (Figure S2A) or other cytokine panels either between CAD patient groups or when compared with the controls.

### 3.3. Monocyte Migration Marker, CCR1, and Plasma Cytokines Were Associated with CAD Related Clinical Parameters

Several pro-inflammatory cytokines (IL-6, IL-1β, IL-8, IL-1RA, TNFα, MCP-1, IL-10, GM-CSF, IL-4, and IL-5) and a monocyte migration marker, CCR1, that were associated with preoperative and postoperative CAD pathogenesis were further analyzed for their association with CAD-related clinical parameters, such as CRP, troponin, lipase, LDH, glucose, creatinine, leukocyte (%), thrombocyte count, body mass index (BMI), and Euroscore (%). Interestingly, we observed that IL-10 and GM-CSF were significantly and positively correlated with troponin (Figure S3A) and TNFα was positively correlated with glucose, whereas CCR1 intensities on classical (M1) and intermediate (M2) monocytes were positively significantly correlated with leukocytes frequencies and were also markedly increased among non-classical monocytes, especially in preoperative CAD patients (Figure S3A). Of note, we did observe increased tendencies for a positive correlation between IL-6 and CRP levels among preoperative patients (Figure S3A). However, cytokine TNF-α was predominant in showing a significant and strong positive association with troponin, glucose, creatinine, and lipase levels (Figure S3B). TNF-α was also positively and significantly associated with the BMI of postoperative CAD patients (Figure S3B). In addition, IL-1β exhibited a significant and positive correlation with leucocytes among postoperative CAD patients (Figure S3B). Furthermore, interestingly, CRP levels exhibited a markedly increased inverse correlation with IL-10 as well as IL-5 cytokines and an increased tendency for a positive correlation with the IL-4 cytokine, especially among postoperative CAD patients.

### 3.4. Effect of Selenium in Minimizing IL-6 and IL-1β Cytokines from Postoperative CAD Mononuclear Cells

**Figure 3:**
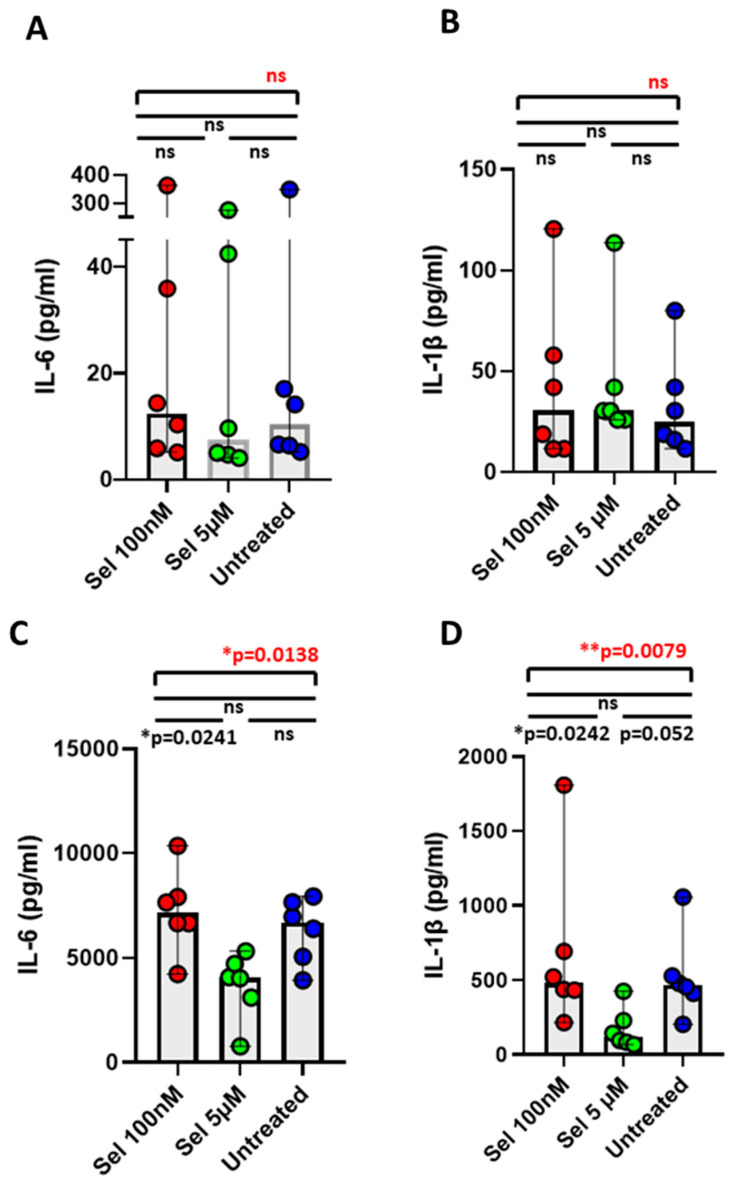
Effect of selenium on levels of IL-6 and IL-1β cytokines among preoperative and postoperative CAD patients. The cell supernatant levels of IL-6 (**A**) and IL-1β (**B**) cytokines from PBMCs of preoperative CAD patients, when incubated (24 h at 37 °C) with two different concentrations of selenium, 100 nM and 5 µM. The cell supernatant levels of IL-6 (**C**) and IL-1β (**D**) cytokines from PBMCs of postoperative CAD patients, when incubated (24 h at 37 °C) with two different concentrations of selenium, 100 nM and 5 µM. Statistical analyses were performed by a non-parametric Kruskal–Wallis test (overall differences between analyzed groups in red) with Dunn´s multiple comparison test (differences between each group in black). The colored dot plots represent individual data values. The vertical lines in the scatter dot plot with bar represents the median with range. (* *p* ≤ 0.05; ** *p* ≤ 0.01; ns: non-significant).

PBMC isolated from six preoperative and six postoperative CAD patients were exposed to two different doses of selenium and were determined for the therapeutic effect of selenium on an array of cytokines and chemokines. Intriguingly, we observed a significant reduction in the cell supernatant level of IL-6 cytokine, with 5 µM selenium in comparison to 100 nM selenium, among postoperative CAD patients (Figure 3C). Likewise, a significant reduction in the cell supernatant IL-1β cytokine with 5 µM selenium in comparison with 100 nM selenium as well as with the untreated group was noticed among postoperative CAD patients (Figure 3D). However, we did not notice any decrement in either the IL-6 (Figure 3A) or IL-1β levels (Figure 3B) in mononuclear cells from preoperative CAD patients. Importantly, selenium did not decrease IL-1RA cytokine in PBMCs from preoperative (Figure S4A) as well as postoperative CAD patients (Figure S4B). Likewise, selenium did not decrease IL-10 cytokine in PBMCs from preoperative (Figure S4A) as well as postoperative CAD patients (Figure S4B). Though we did not observe any reduction in the levels of the rest of the analyzed cytokines and chemokine upon selenium treatment either among preoperative or postoperative CAD patients, we did notice a marked reduction, though not significant, in TNF-α cytokine with 5 µM selenium, especially among postoperative CAD patients.

In addition, we employed a dynamic condition with the Chandler loop system to evaluate the therapeutic effect of selenium (1 µg for 5 mL blood) on the levels of plasma cytokines, post 6 h, 12 h, and 24 h as well as on monocytes’ migration markers, post 6 h and 12 h. However, we did not demonstrate any obvious differences between selenium-treated and untreated groups.

### 3.5. Markedly Lowered Phosphorylation of STAT-3 under the Influence of Selenium in Postoperative CAD Mononuclear Cells

**Figure 4:**
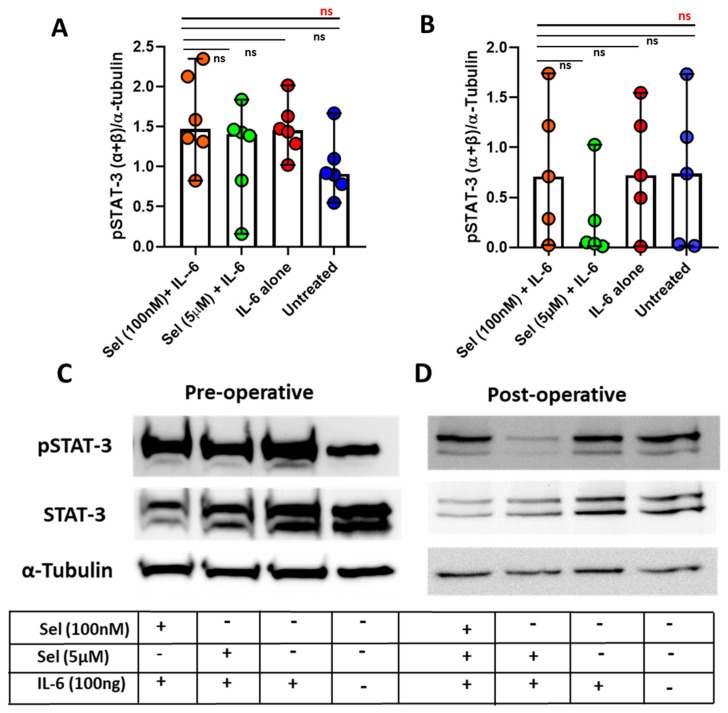
Effect of selenium on phosphorylation of the STAT−3 transcription factor among preoperative and postoperative CAD patients. PBMCs from preoperative (**A**) and postoperative (**B**) CAD patients were incubated with two different concentrations of selenium (100 nM and 5 µM) for 24 h at 37 °C and were subsequently treated with recombinant (r) IL−6 cytokine (100 ng/mL) for the last 30 min of incubation and compared with rIL-6 alone and untreated control. Representative bands of pSTAT−3, STAT−3, and α −tubulin proteins and corresponding treatment strategy for preoperative (**C**) and postoperative (**D**) CAD patients. Statistical analyses were performed by a non-parametric Kruskal–Wallis test (overall differences between analyzed groups in red) with Dunn´s multiple comparison test (differences between each group in black). The colored dot plots represent individual data values. The vertical lines in the scatter dot plot with bar represents the median with range. (ns: non-significant).

PBMCs isolated from six preoperative patients and five postoperative CAD patients were exposed to two different doses of selenium and were stimulated with recombinant IL-6 to determine role of selenium on STAT-3 phosphorylation. Though we did not notice any significant reduction in the phosphorylation of the STAT-3 transcription factor among preoperative CAD patients (Figure 4A,C), we did observe a marked reduction in the phosphorylation status of STAT-3, but it still had not reached the level of statistical significance among postoperative CAD patients (Figure 4B,D).

### 3.6. Curtailed Levels of IL-1β and Reduced Densities of Cleaved Caspase-1 under the Influence of Selenium in Preoperative CAD Mononuclear Cells

**Figure 5:**
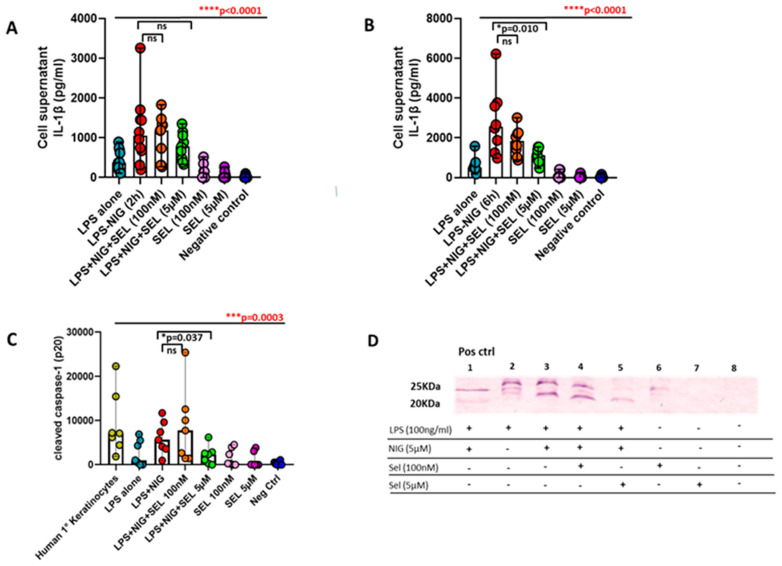
Effect of selenium on pro-inflammatory IL-1β levels and cleaved caspase-1 (p20 subunit) among preoperative CAD patients. PBMCs from preoperative CAD patients were primed with LPS (100 ng/mL) for 16 hrs at 37 °C and were subsequently activated with nigericin (5 µM) for 2 h (**A**) and 6 h (**B**) and incubated with two different concentrations of selenium (100 nM and 5 µM) and compared with selenium treatment (100 nM as well as 5 μM) alone and untreated controls. The cell supernatants levels of IL-1β cytokine (**A**,**B**) and for cleaved caspase-1 protein (p20) secretion were analyzed (**C**). Representative bands for (p20 cleaved caspase-1) protein (**D**) and corresponding treatment strategy for preoperative CAD patients. Statistical analyses were performed by a non-parametric Kruskal–Wallis test (overall differences between analyzed groups in red) with Dunn’s multiple comparison test or by using a non-parametric Mann–Whitney test (differences between each group in black). The colored dot plots represent individual data values. The vertical lines in the scatter dot plot with bar represents the median with range. ((* *p* ≤ 0.05; *** *p* ≤ 0.001; **** *p* ≤ 0.0001; ns: non-significant).

PBMCs obtained from eight preoperative CAD patients were exposed to two different doses of selenium and were also subsequently stimulated with LPS and nigericin to activate the caspase-1 pathway and were further determined for the therapeutic role of selenium on the (i) levels of IL-1β and (ii) cleaved caspase-1 (p20). Though we did not notice any significant reduction in IL-1β levels between 5 µM selenium, LPS, and nigericin for the 2 h treated group alone (Figure 5A), strikingly, we did observe significantly reduced levels of IL-1β with 5 µM selenium in comparison to LPS and nigericin for the 6 h treated group alone (Figure 5B). Furthermore, very interestingly, there was a significant reduction in the densities of cleaved caspase-1 (p20) (N = 7) when treated with 5 µM selenium in comparison to LPS and nigericin for the 6 h treated group alone (Figure 5C,D).

### 3.7. Negligible Effect of Selenium on Human Vascular Endothelial Cells

We further employed human endothelial cells (human umbilical vascular endothelial cells (HUVECS) and/or primary vascular endothelial cells) to evaluate the effect of selenium. Here, selenium-treated endothelial cells were exposed to either LPS or IL-6 to evaluate the phosphorylation status of transcription factors (p65 and STAT-3, respectively) as well as the cell supernatant levels of TNF-α and IL-1β cytokines. We did not observe any effects of selenium. Additionally, we evaluated the effect of selenium (100 nM and 5 µM) on the expression of regulatory-related costimulatory molecules (PD-L1 and ICOS-L) and vWF on pVECs; we did observe tendencies for the increasing intensities of PD-L1 and ICOS-L and tendencies for the decreasing intensities of vWF in the 5 µM selenium-treated group.

## 4. Discussion

To the best of our knowledge, this is the first study demonstrating the differences in the intensities of monocyte migration marker, CCR1, as well as the impact of selenium in mitigating IL-6 and IL-1β cytokine axes at its molecular level, by comparing the preoperative and postoperative cohorts of CAD patients. Since aging is one of the dominant risk factors for the development of atherosclerotic lesions in arteries and CAD etiology [51], in this study, young adults (less than 30 years) were also included (in addition to the older controls), who could presumably be devoid of atherosclerosis, as it has been reported that about 3% of symptomatic CAD occurs below 40 years of age [52]. Nevertheless, the development of atherosclerotic plaques in young adults could be attributed more to risk factors, such as obesity, smokers, and a family history of CAD [53].

Due to the intimate relationship between atherosclerosis underlying CAD and inflammation, blood-circulating monocytes, migration markers, and plasma levels of cytokine and chemokines were studied to assess the state of inflammation among preoperative and postoperative CAD cohorts, also in comparison to the controls. Significant reductions in the frequencies of blood monocytes among postoperative CAD were evident in comparison with preoperative CAD cohorts, which could possibly indicate necroptosis of monocytes due to the high shear stress generated by utilization of the cardiopulmonary bypass (CPB) during surgery [54]. In addition to this, both a reduction in the frequency of classical monocytes (M1) and intensities of the CCR2 migration marker among postoperative CAD patients could either imply their rapid recruitment to atherosclerotic lesions as reported with equivalent mouse monocyte homolog expressing CCR2 (Ly6C^hi^) [55] or might reflect their decrement due to CBP usage. Since the CCR2 receptor is known to possess several ligands (MCP-1, MCP-3, MCP-4) [55,56,57], we cannot exclude the possibility of a “masking effect” by these ligands on CCR2-expressing classical monocytes as a cause for their reduced detection, and certainly all of these explanations require further investigation. Further, the CX_3_CR1 marker that is important for maintaining blood monocyte homeostasis was notably reduced in the classical monocytes of postoperative CAD patients, which could possibly imply infiltration of monocytes to the inflammatory milieu, thereby aggravating inflammation among these patients [58,59]. Strikingly, CCR1-expressing monocytes were significantly increased among the postoperative CAD cohort, in comparison to the preoperative CAD cohort as well as the controls, characterizing a switch from the monocyte to macrophages’ differentiation process, where CCR2 intensities are reported to decline with a substantial increase in CCR1 [60]. Further, our data is in accordance with few reports that evidently show CCR1^high^ monocytes in patients with progressive inflammatory diseases, such as knee osteoarthritis [61], multiple sclerosis [62], systemic sclerosis [63], interstitial lesions of glomerular diseases [64], and hypertension [65].

Though significantly increased levels of IL-6, IL-1RA, and IL-8 cytokines were evident among the preoperative CAD cohort due to underlying chronic inflammation, the magnitude of IL-6 cytokine and IL-1RA were much higher after cardiac bypass surgery, indicating a more pronounced degree of inflammation among the postoperative CAD cohort, which is indeed in accordance with other studies [66,67,68]. Furthermore, among the postoperative CAD cohort, increased levels of IL-4 cytokine could exhibit a pro-inflammatory nature, which might possibly lead to the generation of reactive oxygen species (ROS) and subsequent vascular inflammation, thereby augmenting inflammation [69]. It was also evident from our study that IL-4 levels showed an increased tendency for direct association with CRP levels among postoperative patients. Of note, a decreased secretion of GM-CSF among the postoperative CAD cohort in comparison to the preoperative CAD cohort could possibly aggravate atherosclerosis, as evidenced by accumulating PPAR-γ^low^ macrophages that positively regulate inflammation [70] and cholesterol metabolism [71], and increased lesions and decreased tolerogenic dendritic cells in experimental models, imparting its anti-atherogenic role [72]. Furthermore, elevated levels of IL-5 among the postoperative CAD cohort might possibly explain their involvement in repair mechanisms to tissue injury by promoting eosinophil expansion and polarization towards resolving-type M2 CD206^+^ macrophages, as supported by experimental data [73]. Likewise, an increase in the IL-10 cytokine was more evident among postoperative CAD patients, reflecting the activation of anti-inflammatory responses to counter-balance the escalating inflammation post-surgery [74]. Therefore, it is evident that a substantial increase in both the anti-inflammatory IL-10 cytokine and pro-inflammatory cytokines tend to maintain their balance during amplified inflammation occurring due to cardiac bypass surgery [68]. In fact, our study has also shown a possible regulatory nature of cytokines IL-5 and IL-10, which exhibited a higher tendency for an inverse relationship with CRP among postoperative patients. Though plasma TNFα did not significantly differ between the preoperative and postoperative CAD cohort, postoperative TNFα levels were directly correlated to baseline serum troponin, creatinine, glucose levels, and BMI, which might be a predictor of severity and poor prognosis among postoperative CAD patients [75], who might be comorbid with chronic kidney failure, type 2 diabetes, and obesity. The levels of preoperative anti-inflammatory cytokine IL-10 and possible anti-atherogenic GM-CSF were positively correlated to serum troponin, indicating the existence of attenuating inflammation, succeeding the release of troponin that is yet to be investigated. Regardless, very interestingly, it was shown that the synthesis of the monocytic IL-6 cytokine and STAT-3 phosphorylation is mediated by the CCR1 receptor, which is also linked to secretion of the IL-8 cytokine [76], which demonstrates enhanced IL-6 and IL-8 production and increased monocytic CCR1 expression among the postoperative CAD cohort, corroborating our findings. Our findings undoubtedly confirm the increased state of inflammation and more propensity towards atherosclerotic plaque rupture among postoperative CAD cohorts by demonstrating imbalanced cytokine levels and high-potential macrophage-polarizing CCR1^high^ monocytes.

With these confirmed signature cytokine candidates associated with the increased state of inflammation, we next intend to intervene on the cytokine axes, particularly the IL-6 and IL-1 axes at their molecular level, with selenium. For this purpose, we considered in vitro selenium intervention among two CAD cohorts, representing low-state inflammation (preoperative) and high-state of inflammation (postoperative). Since selenium is inversely associated with CAD risk and IL-6 levels among elderly people [77], we indeed demonstrated a therapeutic effect of selenium in mitigating the IL-6 levels by postoperative mononuclear cells, which was consistent with a report stating reduced IL-6 levels upon treatment with selenium, with different formulations, in the ApoE^−/−^ atherosclerosis mouse model [78]. In addition, selenium has shown a markedly reduced phosphorylation status on the STAT-3 transcription factor in postoperative CAD mononuclear cells. This finding was supported by in vivo experimental models, where selenium substantially reduced phospho-STAT-3 activity on the renal tissue of diabetic nephropathy rats [79] as well as suppressed phospho-STAT-3 nuclear translocation in the cortex microglia of depression-induced mice [80]. On the other hand, few reports have shown a direct relationship between selenium and phospho-STAT-3 activity, where a deficit in selenium in cardiomyocytes exhibited a decline in mitochondrial phosho-STAT-3 activity, leading to a further reduced mitochondrial function and promoting cardiomyocyte apoptosis [81]. However, all these studies involved different cell types and cellular localization of phospho-STAT-3 activity, disease pathologies, and species, and, of course, therapeutic dosage and formulation of selenium, which could explain the reason behind the dual functionalities of selenium on STAT-3 phosphorylation.

Though there were no obvious differences with IL-1β cytokine between the preoperative and postoperative CAD cohorts, the main reason for us to target the IL-1β axis with selenium was that the levels of IL-1β are directly associated with circulating leucocyte frequencies, possibly signifying its pathological functions, including leucocyte endogenous mediators [82] among the postoperative CAD cohort. In addition to this, IL-1RA was significantly increased among the postoperative cohort. Despite a prominent antagonistic role of IL-1RA on pro-inflammatory IL-1 cytokines (IL-1α and IL-1β) by binding to the IL-1 receptor (IL-1R) with high affinity [83], it is also concomitantly secreted from cells almost 4-10 higher fold in comparison to IL-1 cytokines [84], and is therefore considered as a reliable assessment for the production of IL-1-associated cytokines [85]. Intriguingly, in our study, though selenium could significantly attenuate the production of IL-1β by postoperative mononuclear cells, the levels of IL-1RA cytokine were not impeded by selenium, indicating a selective mode of action of selenium on inflammatory IL-1β without impairing the levels of the beneficial antagonistic cytokine, IL-1RA. This mechanism holds true as well for the anti-inflammatory IL-10 cytokine. In fact, increased IL-1RA levels in our study could indicate an already polarized state of monocytes into macrophages [86]. Not only this, but selenium had indeed curtailed the levels of IL-1β as well as the densities of cleaved caspase-1 when preoperative mononuclear cells were induced with inflammasome activators [87]. This finding reflects its potent therapeutic effect on key inflammasome pathways, and our finding is evidently supported by a report showing a lowered aortic gene expression of IL-1β when treated with both sodium selenite and selenium-nanoparticles in the atherosclerosis mouse model [78]. However, this effect was not evident on other diseases, such as gestational diabetes, showing the differences in selenium’s effect on different disease pathologies [88].

Since mononuclear leukocyte-endothelium interaction play a key role in endothelial dysfunction among CAD patients [89], we extended our study to observe the mitigating effects of selenium on an inflammation-induced endothelium. Though there was no prominent effect of selenium on the vascular endothelium with regard to the IL-6/STAT-3 axis, we observed markedly increased intensities of costimulatory molecules, PD-L1 [90] and ICOS-L [91], which are well studied for their immune regulatory functions, but do not reach the level of significance. This might be due to the limitations of a small sample size and the concentration of selenium employed for endothelial cells in the study, which demands further investigation. Henceforth, we cannot exclude the beneficial role of selenium on the vascular endothelium.

Since almost 1 billion people in this world are deficit with the selenium micronutrient [92], a mini cohort-sized clinical study is commendable to determine the increasing kinetics of selenium concentration in the blood of CAD patients and controls, upon dietary selenium supplementation, which might be comparable to the selenium concentration of 5 µM that exhibited a favorable outcome in our in vitro study. Furthermore, in the near future, a clinical study with selenium supplementation before CABG surgery and respective postoperative cytokine profiling and activation in PBMCs and monocytes is needed to confirm our in vitro data.

Taken together, our findings exemplify a high degree of inflammation among postoperative CAD patients compared with preoperative CAD patients, where increased intensities of CCR1-expressing monocytes and production of pro-inflammatory cytokines IL-6, IL-1, and IL-8 were evidently observed. Further, our study has deciphered the molecular activities of cytokine axes through in vitro intervention with selenium, which impeded IL-6/STAT-3 as well as IL-1β/cleaved caspase-1 axes in postoperative and preoperative CAD mononuclear cells. Though further investigations are required to clarify the beneficial role of selenium in minimizing CCR1-expressing monocytes, this study particularly underscores the essentiality for a rapid declining of inflammation, probably with selenium, in order to minimize aggravating atherosclerosis and inflammation-induced damage to the myocardium and bypass grafts, especially among postoperative CAD patients.

## Figures and Tables

**Table 1:** Basic characteristics of preoperative, postoperative, perioperative (vein donor) CAD patients and controls.

Patient Characteristics	CAD (Pre-Op)	CAD (Post-Op)	CAD (Vein Donor)	Control (Young)	Control (Old)
Number of Subjects (*n*)	47	26	4	10	10
Mean Age (years) (Min–Max)	65 (40–82)	71 (59–84)	74 (71–78)	27 (21–39)	56 (50–64)
Gender (men/women)	35/12	17/8	4/0	5/5	3/7
Mean body mass index (Min–Max)	29.4 (21.1–42.4)	28.5 (21.9–35)	27.9 (24.4–30.6)		
CAD patient (1/2/3 vessel disease)	(1/5/41)	(0/2/23)	(0/0/4)		
Mean Euroscore II (%) (Min–Max)	2.57 (0.50–25.85)	2.38 (0.78–5.13)	2.08 (0.94–4.20)		
Acute Infarctions No: STEMI < 1 week STEMI > 1 week NSTEMI < 1 week NSTEMI > 1 week	38 3 1 0 2	15 1 1 0 4	4 0 0 0 0		
Leftventricular Ejection Fraction (LVEF) % (Mean)	52	55	50		

**Table 2:** Clinical characteristics of preoperative, postoperative, and perioperative (vein donor) CAD patients.

Patient Clinical Paramaters	CAD (Pre-Op)	CAD (Post-Op)	CAD (Vein Donor)
***A. Blood parameters***			
Leukocytes (Gpt/L)	9.47	9.37	6.91
Erythrocytes (Tpt/L)	4.53	4.63	4.86
Haemoglobin (mmol/L)	8.45	8.82	9.20
Hematocrit (L/L)	0.40	0.41	0.44
Mean corpuscular volume (MCV) (fL)	88.97	90.09	91.18
Mean corpuscular hemoglobin (MCH) (fmol)	1.87	1.91	1.90
Mean corpuscular hemoglobin concentration (MCHC) (mmol/L)	20.99	21.2	20.80
Thrombocyte count (Gpt/L)	240.57	241.54	205.25
Proportion, large platelets (%)	30.90	31.12	27.20
Mean thrombocyte volume (fL)	10.75	10.70	10.23
***B. Clinical chemistry parameters (plasma)***			
Troponin T (ng/mL)	0.16	0.11	0.02
Sodium (mmol/L)	139.06	140.95	140.50
Potassium (mmol/L)	4.09	3.93	4.39
Creatinine (µmol/L)	110.27	96.62	85.25
Glucose (mmol/L)	7.34	7.65	8.36
Urea (mmol/L)	6.33	5.58	6.88
Alanine aminotrasferase (ALAT) (µmol/s.L)	0.45	0.54	0.42
Asparate amnotransferase (ASAT) (µmol/s.L)	0.43	0.53	0.40
Creatinine kinase (µmol/s.L)	1.84	2.98	2.33
Lipase ((µmol/s.L))	0.68	0.69	0.64
Cholinestearse ((µmol/s.L))	138.63	132.8	140.75
Lactate dehydrogenase (LDH) (µmol/s.L)	3.56	3.34	3.61
C-reactive protein (CRP) (mg/L)	8.65	5.54	4.90
***C. Clotting parameters***			
Thromboplastin time (%)	95.30	92.29	95.25
International normalized ratio	1.03	1.09	1.04
Activated partial thromboplastin clot time (s)	29.83	34.55	27.98

**Table 3:** Treatment taken prior and after surgery in preoperative and postoperative patient cohort.

Type of Medications	Preoperative Cohort (N)	Postoperative Cohort (N)
**A. Regular Medications**		
Statins	32	15
Beta blockers	35	11
Oral antidiabetics	17	9
Calcium antagonists	16	3
AT_2_ receptor antagonists	20	6
Angiotensin converting enzyme (ACE) inhibitors	18	9
Insulin	5	3
Acetylsalicylic acid (ASA)	43	15
Oral antiplatelet therapy (other than ASA)	6	6
Oral anticoagulation	7	2
Thyroid drugs	11	2
Diuretics	14	4
Antibiotics	0	0
**B. In-hospital Medications (before surgery)**		
Proton Pump Inhibitors (PPI)	All	All
Heparin	All	All
Antibiotics	None	None

N = Number of CAD patients.

## Data Availability

The data presented in this study are available on request from the corresponding author.

## Supplementary Materials

The following supporting information can be downloaded at: https://www.mdpi.com/article/10.3390/ijms24087198/s1.
