# Supplementary material for: Immunophenotyping of Monocyte Migration Markers and Therapeutic Effects of Selenium on IL-6 and IL-1β Cytokine Axes of Blood Mononuclear Cells in Preoperative and Postoperative Coronary Artery Disease Patients
Source: Int J Mol Sci. 2023 Apr 13;24(8):7198. doi: 10.3390/ijms24087198 (PMC10139122; doi:10.3390/ijms24087198)
Supplement: Supplementary file 1 [file ijms-24-07198-s001.zip › REVISED_SUPPLEMENTARY FIGURE LEGENDS..pdf]

SUPPLEMENTARY FIGURE LEGENDS

Figure 1. IL-1β gene expression, FACS plot for monocyte subsets and comparative analysis of intensities of monocyte markers between preoperative and postoperative CAD patients. (A) In vitro IL-1β gene expression in LPS stimulated THP-1 (monocytic cell lines) with different doses of selenium. **(B)** Representative FACS plot for monocyte differentiating into classical, intermediate and non-classical subsets based on CD14 and CD16 markers. **(C)** Intensities of CX_3CR1 on classical monocytes (M1) between postoperative CAD patient and controls. **(D)** Comparative analysis of total blood monocytes frequencies and intensities of CCR2 markers on classical (M1) monocytes and intensities of CCR1 markers on classical (M1), intermediate (M2) and non-classical (M3) monocytes, between preoperative and postoperative CAD patients. Statistical analyses were performed by non-parametric Kruskal-Wallis test (overall differences between analyzed groups, in red) with Dunn´s multiple comparison test or Mann-Whitney test (differences between each group, in black). The vertical lines in scatter plot with bar represents the median with range.

Figure 2. Plasma pro-inflammatory cytokines between preoperative and postoperative CAD patients. (A) Plasma levels of TNF-α between preoperative and postoperative CAD patients and controls. **(B)** Plasma levels of GM-CSF, IL-6, IL-RA, IL-4, IL-5, IL-10 between preoperative and postoperative CAD patients. Statistical analyses were performed by non-parametric Kruskal-Wallis test (overall differences between analyzed groups, in red) with Dunn´s multiple comparison test or Mann-Whitney test (differences between each group, in black). The vertical lines in scatter plot with bar represents the median with range.

Figure 3. **Association between plasma cytokines and monocyte migration marker and clinical parameters among preoperative and postoperative CAD patients. (A)** Positive correlation between cytokines, IL-10 and GM-CSF and troponin; positive tendency between IL-6 and CRP; and positive correlation and tendency between CCR1 migration marker expressed on M1 and M2 and M3 monocyte subsets and leukocytes, respectively, among preoperative CAD patients. **(B)** Positive correlation between the cytokine TNF-α and troponin, creatinine, glucose, lipase and BMI and positive correlation between IL-1β and leukocytes, among postoperative CAD patients. Statistical analyses were performed by non-parametric Spearman´s correlation co-efficient rank test.

Figure 4. Effect of selenium on IL-1RA and IL-10 among preoperative and postoperative CAD patients. The cell supernatant levels of IL-1RA and IL-10 cytokines from PBMCs of preoperative **(A)** as well as postoperative **(B)** CAD patients, when incubated (24hrs at 37ºC) with two different concentrations of selenium, 100nM and 5µM. Statistical analyses were performed by non-parametric Kruskal-Wallis test (overall differences between analyzed groups, in red) with Dunn´s multiple comparison test (differences between each group, in black). The vertical lines in scatter plot with bar represents the median with range.
