# Supplementary material for: Immunophenotyping of Monocyte Migration Markers and Therapeutic Effects of Selenium on IL-6 and IL-1β Cytokine Axes of Blood Mononuclear Cells in Preoperative and Postoperative Coronary Artery Disease Patients
Source: Int J Mol Sci. 2023 Apr 13;24(8):7198. doi: 10.3390/ijms24087198 (PMC10139122; doi:10.3390/ijms24087198)
Supplement: Supplementary file 1 [file ijms-24-07198-s001.zip › REVISED_SUPPLEMENTARY FIGURES.pptx]

## Slide 1
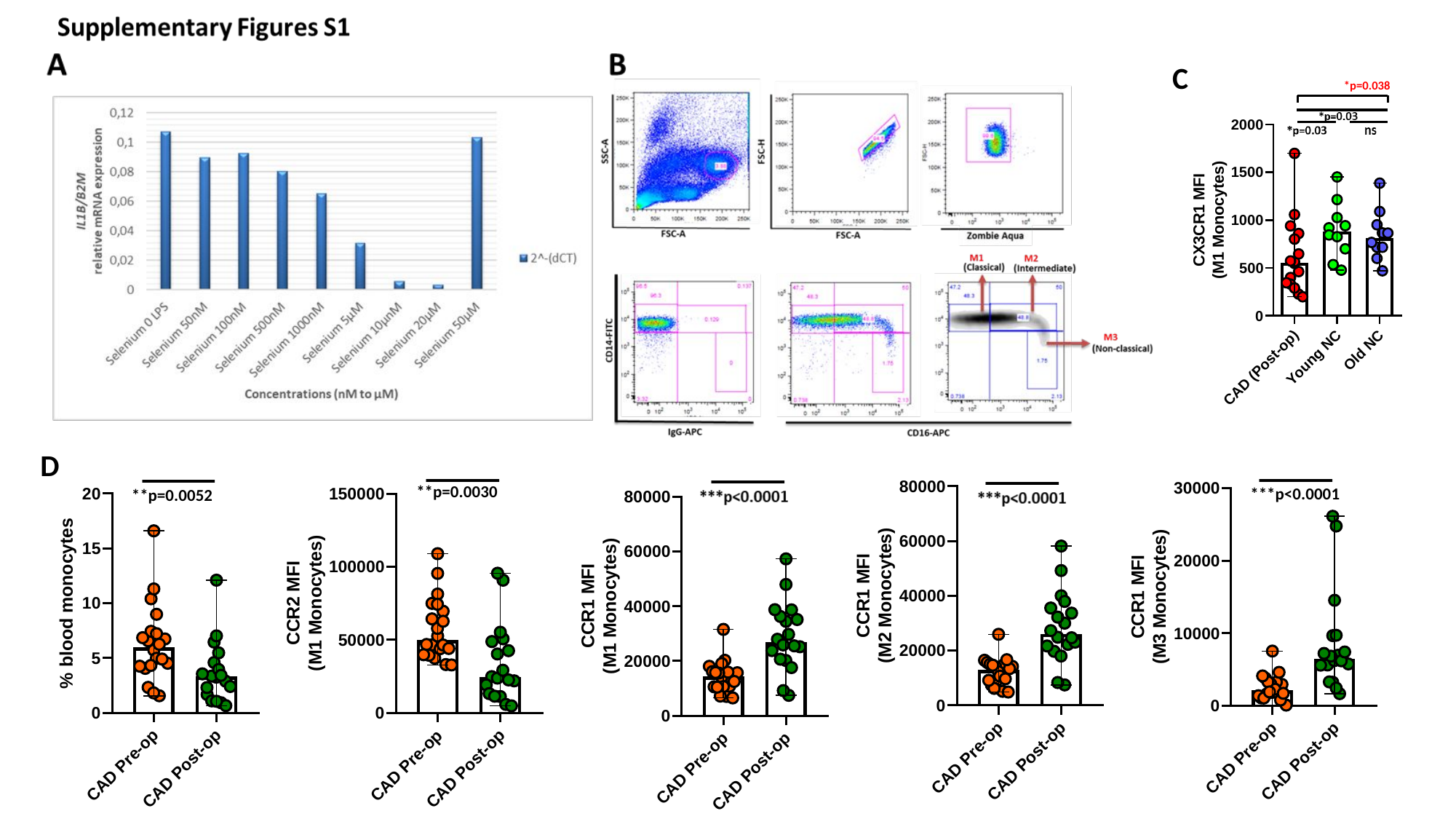

C
*p=0.038
D
**p=0.0030
***p<0.0001
**p=0.0052

## Slide 2
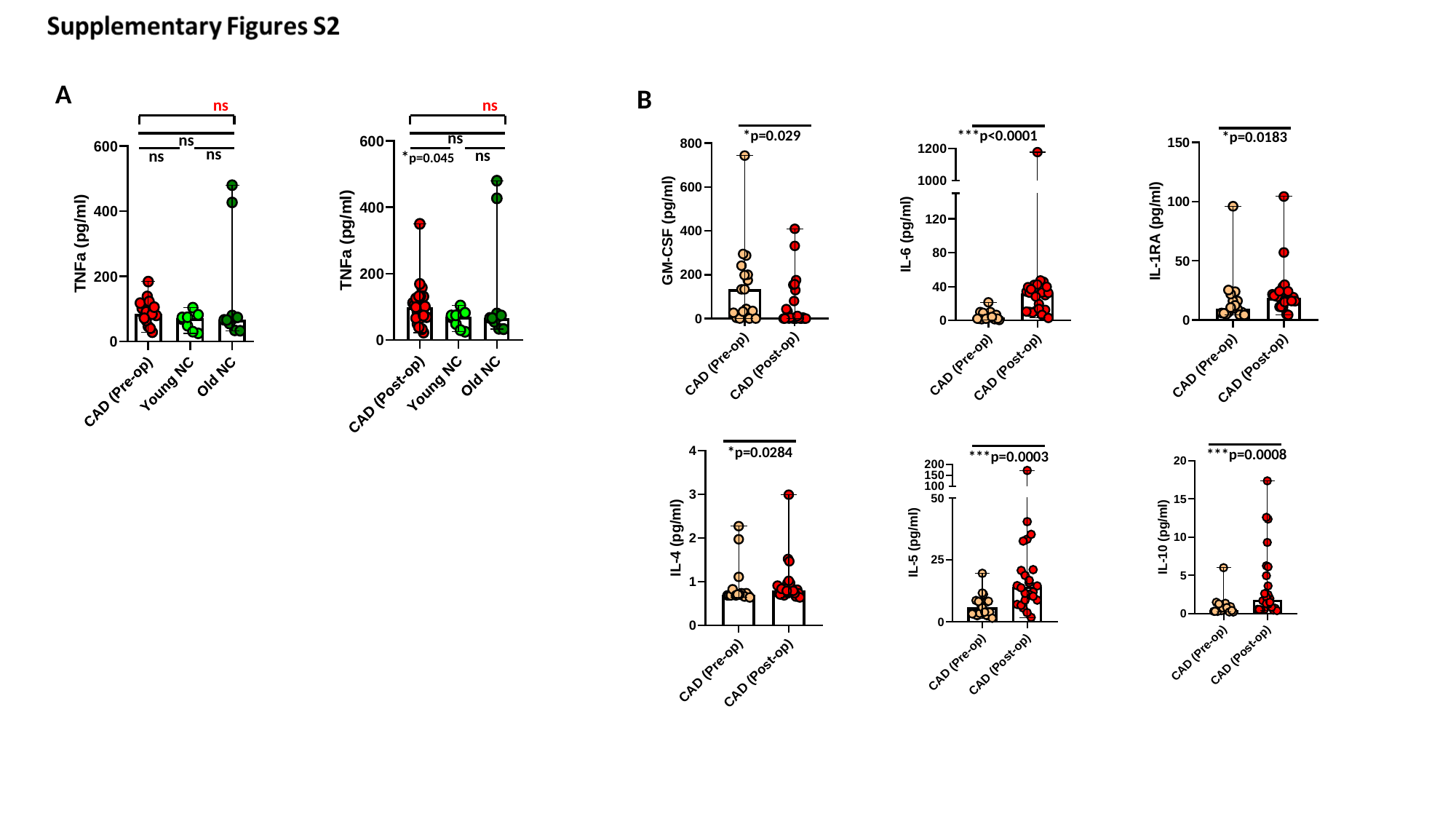

A
B
ns
ns
ns
*p=0.045
ns
ns
ns
ns
*p=0.029
***p<0.0001
*p=0.0183
*p=0.0284
***p=0.0008
***p=0.0003

## Slide 3
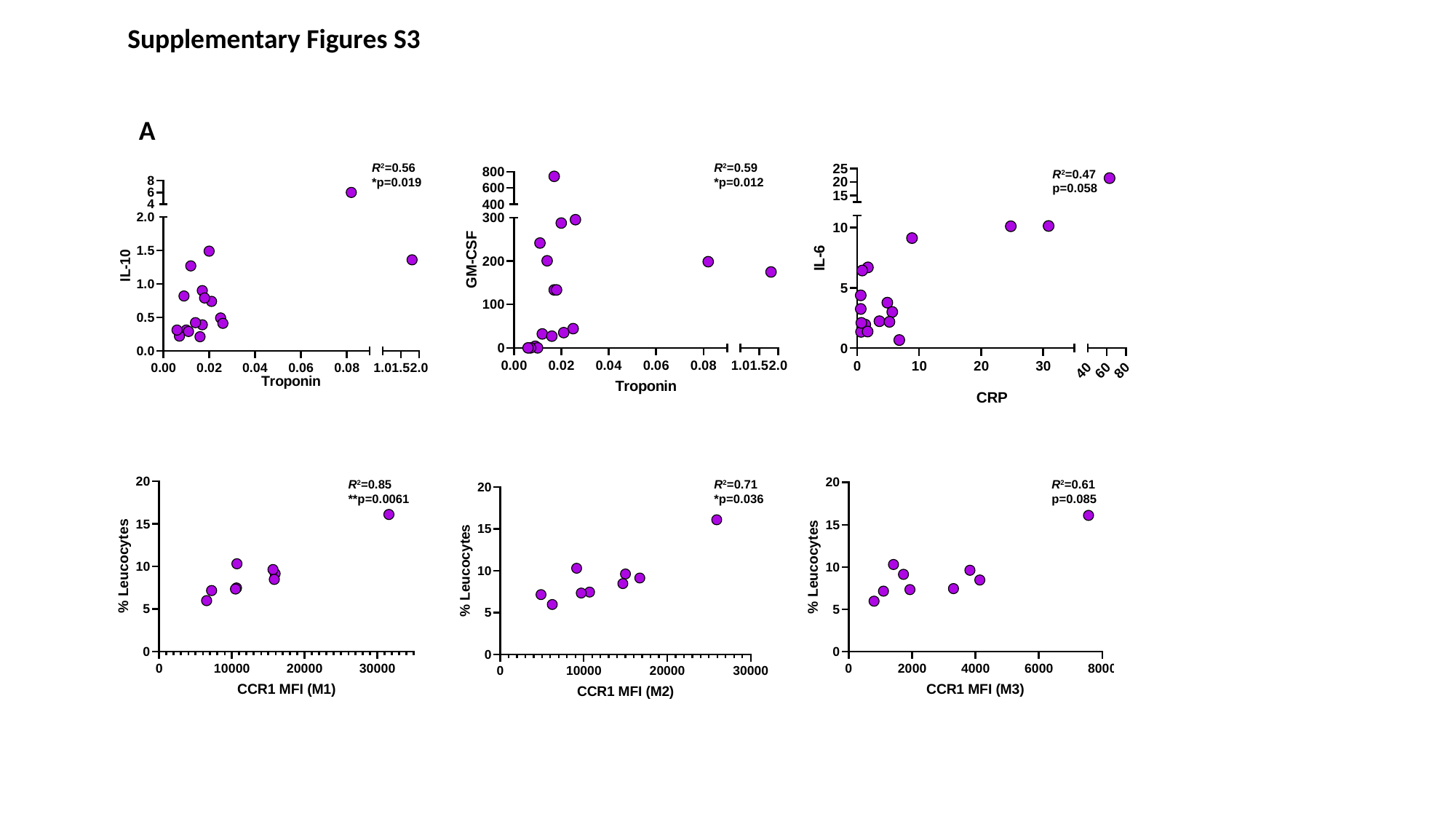

Supplementary Figures S3
A
R2=0.56
*p=0.019
R2=0.59
*p=0.012
R2=0.47
p=0.058
R2=0.85
**p=0.0061
R2=0.71
*p=0.036
R2=0.61
p=0.085

## Slide 4
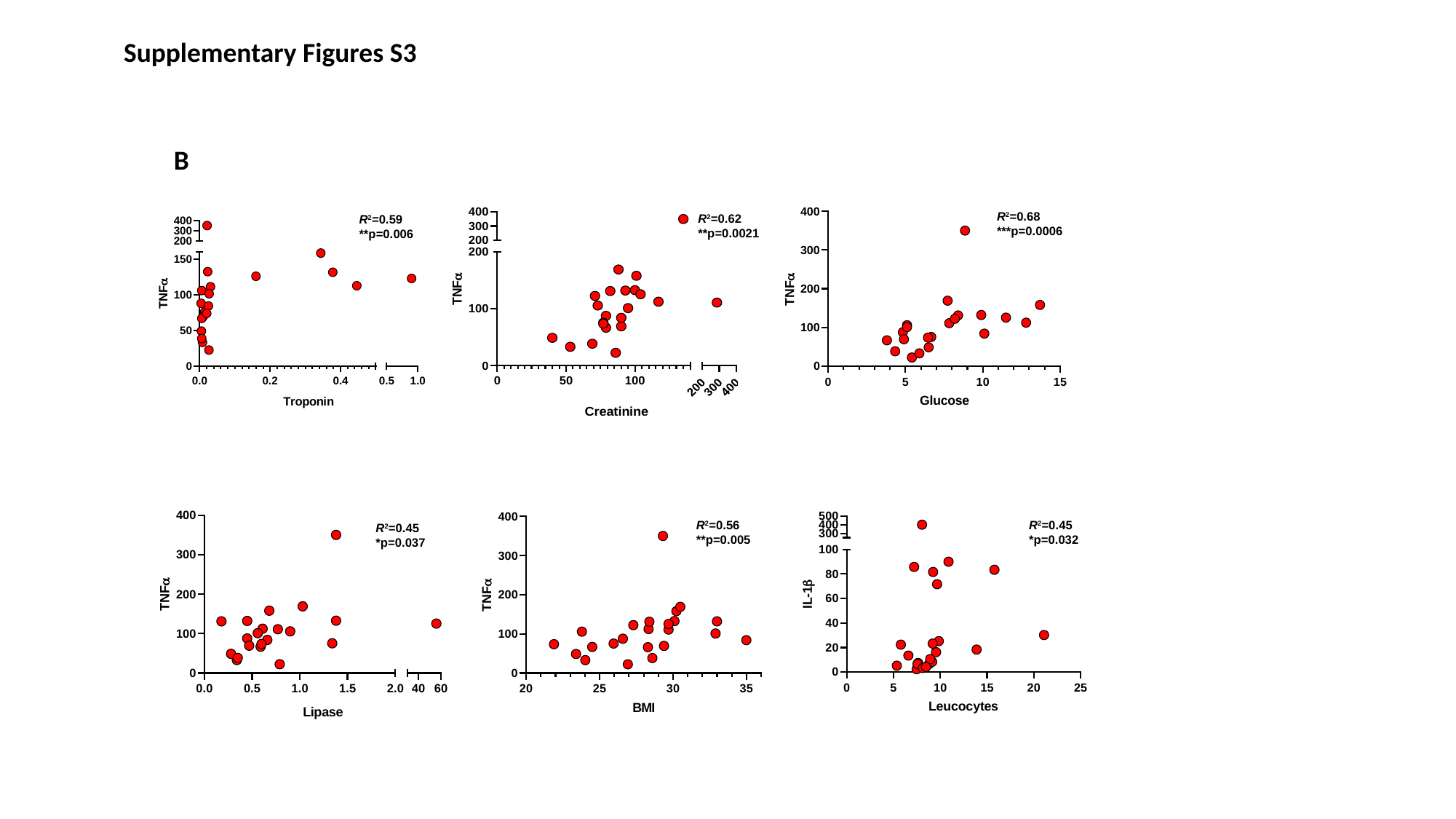

Supplementary Figures S3
B
R2=0.62
**p=0.0021
R2=0.68
***p=0.0006
R2=0.59
**p=0.006
R2=0.45
*p=0.032
R2=0.56
**p=0.005
R2=0.45
*p=0.037

## Slide 5
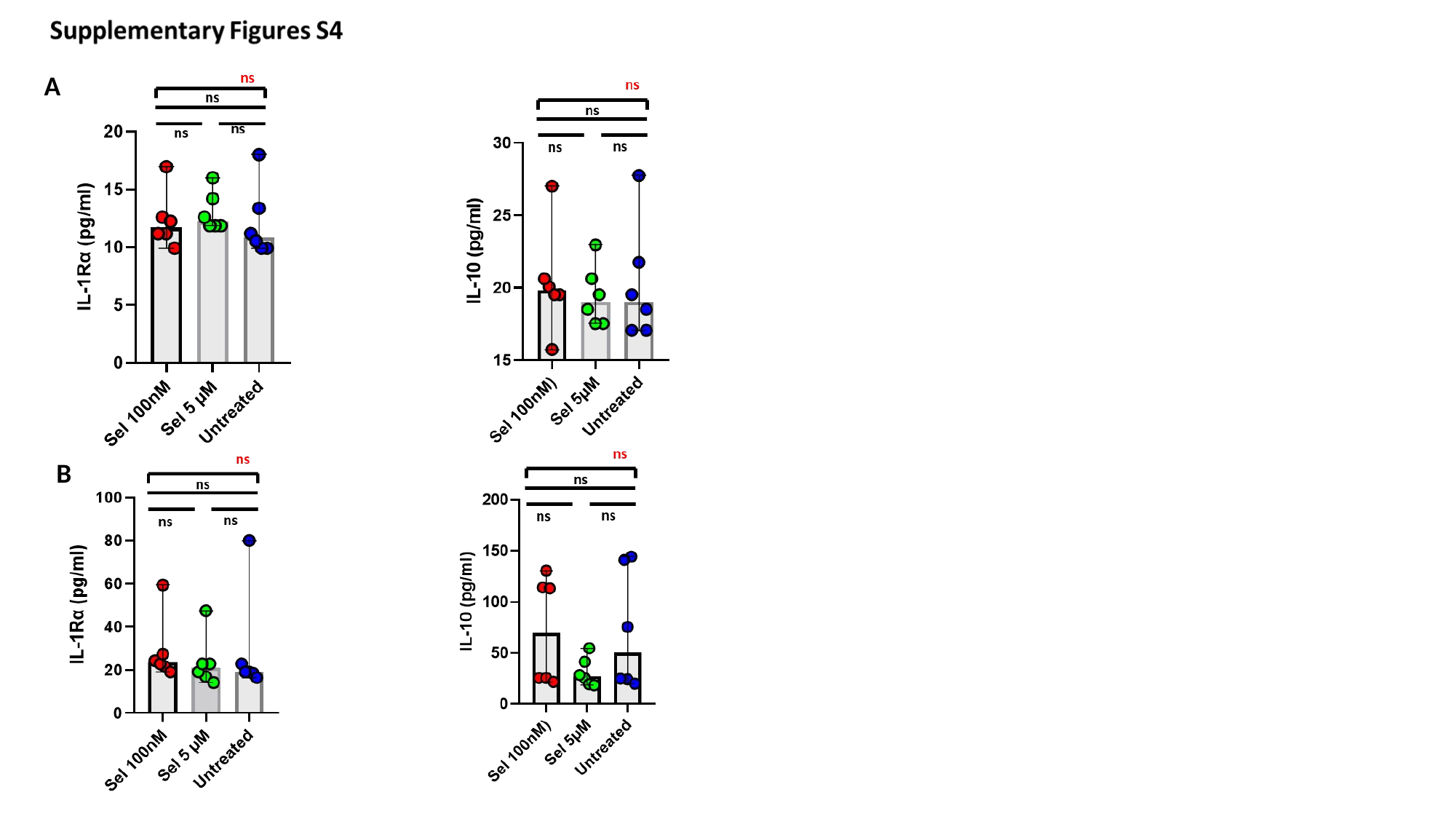

A
B
